# Supplementary material for: Microwave quantum diode
Source: Nat Commun. 2024 Jan 20;15:630. doi: 10.1038/s41467-024-44908-w (PMC10799849; doi:10.1038/s41467-024-44908-w)
Supplement: Supplementary file 1 — Supplementary Information [file 41467_2024_44908_MOESM1_ESM.pdf]

Microwave quantum diode: Supplementary notes

Rishabh Upadhyay[1],* Dmitry S. Golubev[1], Yu-Cheng Chang[1], George Thomas[1,2], Andrew Guthrie[1], Joonas T. Peltonen[1], and Jukka P. Pekola[1]

[1] *Pico group, QTF Centre of Excellence, Department of Applied Physics, Aalto University School of Science, P.O. Box 13500, 00076 Aalto, Finland and*

[2] *VTT Technical Research Centre of Finland Ltd, Tietotie 3, 02150 Espoo, Finland*

SUPPLEMENTARY NOTE 1

Simulations. We use lumped elements circuit simulator (QUCS), and high-frequency RF/MW electromagnetic analysis (SONNET), to simulate the two meandering wire shunted resonators to ensure the resonances we measured are reasonable, as shown in Supplementary Fig. 1. The lengths of the CPW waveguide part of the resonators are 4620 um on the left and 4007 um on the right. The corresponding inductance of the left (L_1) and right (L_2) meandering wire is ≈ 0.1 nH and ≈ 0.25 nH [1]. From finite element analysis simulator (COMSOL Multiphysics), we calculate the coupling capacitance value of 6.98 fF between the transmission line and the resonator (C_{left} and C_{right}), which we use in simulations. The simulated resonance frequencies from QUCS were 6.072 GHz and 6.394 GHz. Simulations from Sonnet reveals the frequencies of the center lines at 5.311 GHz and 6.130 GHz. Since in the reported device we use air bridges to connect the ground planes around the circuit, therefore we now repeat the simulation in Sonnet using the air bridges. In superconducting quantum devices, air bridges [2, 3] holds the balance of the ground planes around the central lines, reduces the possibility of microwave loss due to mode mixing, and avoids pseudo resonances. The simulated designs with and without air bridges are reported in Supplementary Fig. 1 (b,c). After adding the air bridges to mimic the reported device, the simulated frequencies rise to 5.706 GHz and 6.182 GHz. Both of these frequencies are lower than the designed bare frequencies of the left and right resonators, this could be due to the imperfect isolation of the propagating microwave. We observed that the simulated current-field distribution at the resonance frequency at one side of the device shows a part of the field leaking across the other side because of the imbalance of the grounding around the meandering shunt that could make the wave to propagate along the ground surrounded by the centre line of the off-resonance resonator. This imbalance issue could be verified when we placed a perfect conductive block as shown in Supplementary Fig. 1 (d), between the meandering wire to connect the upper (ground 1) and lower (ground 2) ground planes of the device, and as a result we observed that the frequencies rise to the values very close to QUSC. The experimentally observed lower frequency (6.027 GHz) is within a reasonable range obtained from Sonnet and QUCS simulations, but the higher frequency (6.762 GHz) is higher than the simulated frequency from QUCS. As shown in Supplementary Fig. 1 (a), adding an inductor with around 0.5 nH between the floating sides of the meandering wires to mimic the Josephson junction could increase the frequency to 6.7 GHz. Hence, both of our measured frequencies are under the reasonable range and are used in Appendix for calculations.

* rishabh.upadhyay@aalto.fi

SUPPLEMENTARY NOTE 2

Theory. In this Appendix we briefly present the theory of the transmission rectification effect in our device. The system is described by the Hamiltonian

$$H = H_{\rm res} + H_{\rm loop} + H_{\rm int}. \tag{1}$$

The Hamiltonian of the two resonators is given by

$$H_{\rm res} = \sum_{j=1}^{2}\left[\hbar\omega_j a_j^\dagger a_j + \sqrt{\frac{\hbar\kappa_{cj}Z_0}{\omega_j^3}}\frac{dI_j(t)}{dt}(a_j^\dagger + a_j)\right] - \hbar g_{12}(a_1^\dagger + a_1)(a_2^\dagger + a_2), \tag{2}$$

where $\omega_j = 2\pi f_j$ are the angular frequencies of the fundamental modes of the resonators 1 and 2 (in our sample $\omega_2 > \omega_1$), a_j are the corresponding ladder operators, κ_{c1}, κ_{c2} are the damping rates of the resonators due to their capacitive coupling to the transmission lines,

$$\kappa_{cj} = \frac{2\omega_j^3 Z_0^2 C_{Kj}^2}{\pi}, \tag{3}$$

Z_0 is the resonator impedance, C_{K1}, C_{K2} are the capacitors connecting the resonators to the transmission lines (see Fig. 1 (c) in the main text), $I_1(t), I_2(t)$ are the input microwave currents which are related to the incoming powers as $P_j^{\rm in} = Z_0\overline{I_j^2(t)}$ (here bar implies the time averaging), and g_{12} is the coupling strength between the two resonators.

This coupling is mediated by the inductances of the coupling elements L_1, L_2, by the total inductance of the upper part of the SQUID loop containing the three junctions L_q, and by the total capacitance of the Josephson junctions C_q, see Fig. 1(c) of the main text and Supplementary Fig. 1(a). The direct coupling between the resonators g_{12} is not sensitive to the state of the qubit.

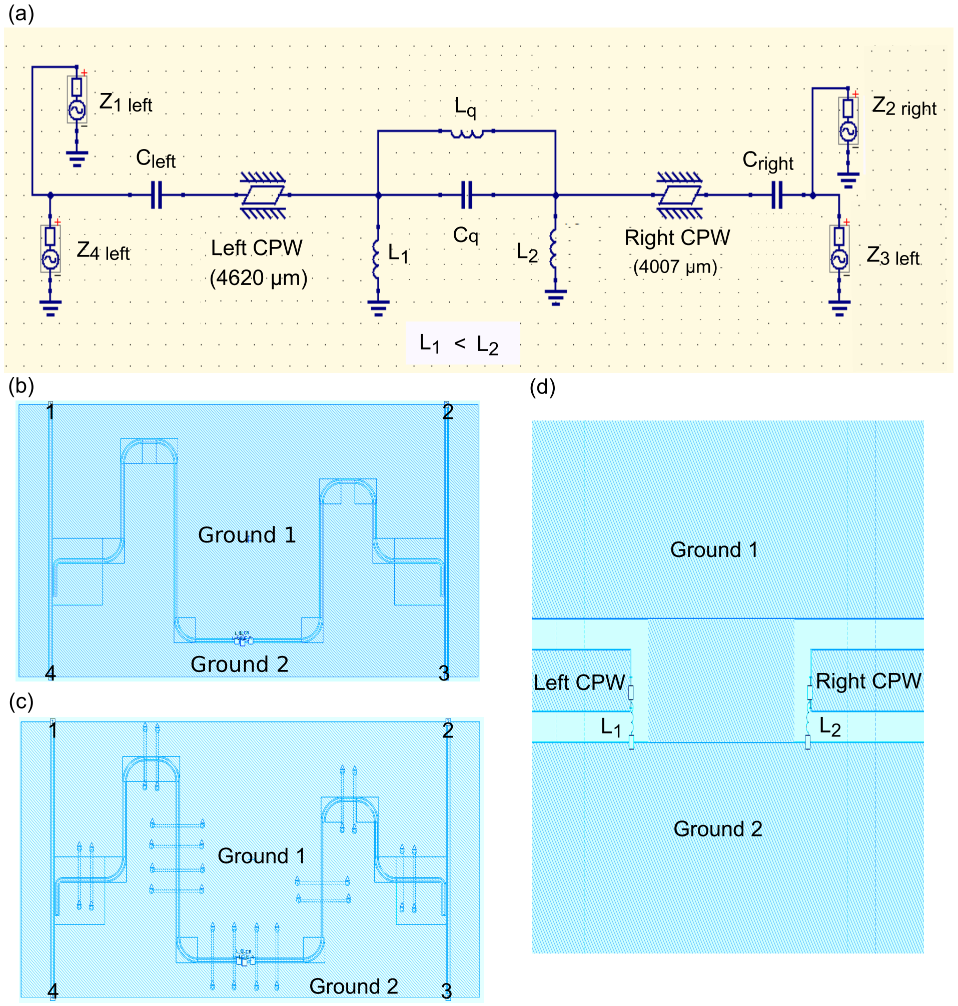

Supplementary Figure 1. Device simulations (a) Simulated circuit design image from QUCS, multiports network analysis. (b) Simulated design image using Sonnet with no bonding wires. (c) Al Bonding wires mimicking the real device in panel to connect upper (ground 1) and lower (ground 2) grounds. (d). A metallic block to connect the upper and lower ground planes.

In the weak coupling limit g_{12} can be approximately estimated as

$$g_{12} \approx \frac{2}{\pi} \frac{\omega_1 \omega_2 L_1' L_2'}{Z_0} \left(C_q \omega_1 \omega_2 + \frac{1}{L_q} \right), \tag{4}$$

although this expression becomes inaccurate if g_{12} becomes comparable to the frequency difference $|\omega_1 - \omega_2|$. The Hamiltonian (Supplementary Eq. (2)) contains the terms proportional to the derivatives of the input currents dI_j/dt, which have been derived within the usual input-output formalism for the resonators [4] without applying rotating wave approximation.

The Hamiltonian of the superconducting loop H_{loop} is expressed in terms of the ladder operators $b, b^\dagger$ describing its low-frequency mode, and the flux dependent qubit frequency $\omega_0(\Phi) = 2\pi f_{01}(\Phi)$,

$$H_{\text{loop}} = \hbar\omega_0(\Phi) b^\dagger b - \frac{E_C}{12}(b^\dagger + b)^4. \tag{5}$$

Here E_C is the effective charging energy of the low frequency mode of the loop, which determines the anharmonicity of the qubit. From the spectroscopic measurements presented in Supplementary Fig. 2 (e,f) we estimate $E_C/(2\pi\hbar) \approx 200$ MHz. Finally, the last term in the Hamiltonian (Supplementary Eq. (6)) describing the interaction between the loop and the resonators has the form

$$H_{\text{int}} = -\hbar g_1 (a_1^\dagger + a_1)(b^\dagger + b) - \hbar g_2 (a_2^\dagger + a_2)(b^\dagger + b), \tag{6}$$

where g_1 and g_2 describe the coupling between the qubit and the corresponding resonator.

We diagonalize the Hamiltonian of the two resonators (Supplementary Eq. (2)) and introduce the hybrid modes with frequencies

$$\omega_{h,l} = \sqrt{\frac{\omega_1^2 + \omega_2^2 \pm \sqrt{(\omega_2^2 - \omega_1^2)^2 + 16 g_{12}^2 \omega_1 \omega_2}}{2}}. \tag{7}$$

In the transmission rectification experiment we probe the range of frequencies close to $f_h = \omega_h/(2\pi) = 6.762$ GHz. For this reason, we leave only the high frequency mode

and write the Hamiltonian of the resonators (Supplementary Eq. (2)) and the interaction term (Supplementary Eq. (6)) in the form

$$H_{\rm res} = \hbar\omega_h a_h^\dagger a_h + \left(\frac{\sqrt{\kappa_{h1}}}{\omega_1^2}\frac{dI_1(t)}{dt} + \frac{\sqrt{\kappa_{h2}}}{\omega_2^2}\frac{dI_2(t)}{dt}\right)\sqrt{\hbar\omega_h Z_0}(a_h^\dagger + a_h), \qquad (8)$$

$$H_{\rm int} = -\hbar g_h(a_h^\dagger + a_h)(b^\dagger + b). \qquad (9)$$

Here $a_h, a_h^\dagger$ are the ladder operators of the hybrid mode with the angular frequency $\omega_h = 2\pi f_h$, κ_{h1} and κ_{h2} are the partial contributions to the total damping rate of this mode coming from the coupling of the resonators 1 and 2 to the transmission lines,

$$\kappa_{h1} = \frac{\omega_1}{\omega_h}\sin^2\theta\,\kappa_{c1}, \quad \kappa_{h2} = \frac{\omega_2}{\omega_h}\cos^2\theta\,\kappa_{c2}, \qquad (10)$$

and the angle θ is determined by

$$\sin 2\theta = \frac{4g_{12}\sqrt{\omega_1\omega_2}}{\sqrt{(\omega_2^2-\omega_1^2)^2 + 16g_{12}^2\omega_1\omega_2}}. \qquad (11)$$

The total damping rate of the hybrid mode is $\kappa_h = \kappa_{h1} + \kappa_{h2} + \kappa_{hi}$, where κ_{hi} describes the internal damping in the resonators and in the qubit, i.e. $\kappa_{hi} = T_1^{-1} + \kappa_{r1} + \kappa_{r2}$, where T_1 is the relaxation time of the qubit and κ_{r1}, κ_{r2} describe the internal damping in the resonators. The couplings to the high frequency mode, g_h, and to the low frequency one, g_l, are expressed as

$$g_h = -(\omega_1/\omega_h)^{1/2}g_1\sin\theta + (\omega_2/\omega_h)^{1/2}g_2\cos\theta,$$
$$g_l = (\omega_1/\omega_l)^{1/2}g_1\cos\theta + (\omega_2/\omega_l)^{1/2}g_2\sin\theta, \qquad (12)$$

Based on Supplementary Eq. (8) we observe that the input currents $I_1^{\rm in}$ and $I_2^{\rm in}$ have different pre-factors. This difference is the origin of the asymmetry in our system. From this pre-factors we can determine the ratio of the powers P_1^* and P_2^*, above which single transmission lines in the coefficients $|S_{31}|^2$ and $|S_{42}|^2$ split into two lines, without solving the problem. Namely, we find

$$\frac{P_1^*}{P_2^*} = \frac{\omega_1^4\kappa_{h2}}{\omega_2^4\kappa_{h1}}. \qquad (13)$$

The powers P_1^* and P_2^* are not equal because the frequencies of the two resonators f_1 and f_2 and/or the couplings between the hybrid mode and the two transmission lines, κ_{h1} and κ_{h2}, differ from each other. This makes our device a non-linear system with asymmetric coupling to the two ports. According to the theory, exactly these properties are required for transmission rectification, see e.g. Ref. [5]. In our device the coupling capacitors C_{K1} and C_{K2} are nominally equal, $C_{K1} = C_{K2}$. Therefore the ratio (Supplementary Eq. 13) can be simplified to $P_1^*/P_2^* = \cot^2\theta$. In the experiment we find $P_1^*/P_2^* = 3.2$, see Fig. 4 in the main text, which corresponds to $\theta = 0.51$. To obtain this value from Supplementary Eq. (11) and, at the same time, to reproduce the experimentally observed frequencies of the modes $f_h = 6.762$ GHz and $f_l = 6.026$ GHz from Supplementary Eq. (7), we choose $f_1 = 6.209$ GHz, $f_2 = 6.595$ GHz and $g_{12}/(2\pi) = 313$ MHz. The simulations reported in Appendix show that these parameters are reasonable. Moreover, with the parameters given in the Appendix A, $L_1 = 0.1$ nH, $L_2 = 0.25$ nH, $L_q = 0.5$ nH and $C_q \approx 100$ fF, Supplementary Eq. (4) gives $g_{12}/(2\pi) \approx 200$ MHz, which is not too far from the estimate given above. To verify our model further, we have estimated the values of the coupling capacitors in COMSOL and found $C_{K1} = C_{K2} = 7$ fF. Adopting this value and the parameters given above, from Supplementary Eq. (3) we obtain $\kappa_{c1}/(2\pi) = 732.6$ kHz, $\kappa_{c2}/(2\pi) = 878$ kHz, and from Supplementary Eq. (10) we find $\kappa_{h1}/(2\pi) = 160$ kHz, $\kappa_{h2}/(2\pi) = 653$ kHz. The damping rates κ_{h1}, κ_{h2} can be independently estimated by fitting the transmission coefficients $|S_{41}|^2$ and $|S_{32}|^2$ at zero magnetic flux, where the qubit is decoupled from the resonator, to the expressions resulting from our model Hamiltonian (Supplementary Eq.(2)):

$$|S_{41}|^2 = 1 - \frac{\omega_h^4}{\omega_1^4}\frac{\kappa_{h1}^2 + 2\kappa_{h1}\kappa_{h2}}{4(\omega-\omega_h)^2 + \kappa_h^2},$$
$$|S_{32}|^2 = 1 - \frac{\omega_h^4}{\omega_2^4}\frac{\kappa_{h2}^2 + 2\kappa_{h1}\kappa_{h2}}{4(\omega-\omega_h)^2 + \kappa_h^2}. \qquad (14)$$

Such fitting procedure gives $\kappa_{h1}/(2\pi) = 160$ kHz, $\kappa_{h2}/(2\pi) = 430$ kHz and $\kappa_h/(2\pi) = 787$ kHz. While κ_{h1} agrees with the theoretical estimate given above, the experimental rate κ_{h2} is a bit lower than the theoretical prediction. With these parameters the ratio of the powers (Supplementary Eq.(13)) becomes 2.1, which is still not very far from the result of the measurements. Thus, we have confirmed that our observations reasonably well agree with the model.

The dependence of the transmission coefficients $|S_{31}|^2$ and $|S_{42}|^2$ on power (see main text Fig. 4) can be understood as follows. At sufficiently low power we can approximately replace the two resonators and the qubit by a single non-linear system with the Hamiltonian

$$H = \hbar\omega_r a^+ a + \frac{\hbar K}{6}(a^\dagger + a)^4 + 2\hbar(\epsilon_1 + \epsilon_2)\cos\omega t(a^\dagger + a). \qquad (15)$$

Here $\omega_r = \omega_h + 2\pi\chi$ is the frequency of the hybrid mode shifted due to the interaction with the qubit, K is the Kerr non-linearity of the combined system and

$$\epsilon_j = \frac{\omega_h^2}{\omega_j^2}\sqrt{\frac{\kappa_{hj}P_j^{\rm in}}{2\hbar\omega_h}}. \qquad (16)$$

It has been experimentally shown that this approximation well describes systems similar to ours [6, 7]. To find the the transmission coefficient $|S_{31}|^2$ at low power we put $\epsilon_2 = 0$ and construct the following ratio:

$$|S_{31}|^2 = \frac{P_3}{P_1^{\rm in}} = \frac{\kappa_{h2}\hbar\omega_r}{2P_1^{\rm in}}\langle a^\dagger a\rangle. \qquad (17)$$

Here P_3 is the power coming to the port 3 and the factor 2 in the denominator accounts for the equal splitting of the power coming out of the resonator 2 between the ports 2 and 3, i.e. we put $\kappa_{h2}\hbar\omega_r\langle a^\dagger a\rangle = P_2+P_3$ and assume that $P_2 = P_3$. The average value $\langle a^\dagger a\rangle$ for the Hamiltonian (Supplementary Eq.(15)) has been evaluated in Ref. [8]. Based on this result we obtain the expression for the transmission coefficient in the form

$$|S_{31}|^2 \approx \frac{\kappa_{h1}\kappa_{h2}}{4(\omega-\omega_r)^2+\kappa_h^2} \times \frac{\left|{}_0F_2\left(1-\frac{\omega-\omega_r-i\frac{\kappa_h}{2}}{K}, -\frac{\omega-\omega_r+i\frac{\kappa_h}{2}}{K}, \frac{2\kappa_h^2 P_1^{\rm in}}{9K^2P_1^*}\right)\right|^2}{\left|{}_0F_2\left(-\frac{\omega-\omega_r-i\frac{\kappa_h}{2}}{K}, -\frac{\omega-\omega_r+i\frac{\kappa_h}{2}}{K}, \frac{2\kappa_h^2 P_1^{\rm in}}{9K^2P_1^*}\right)\right|^2}, \quad (18)$$

where ${}_0F_2(x)$ is the generalized hyper-geometric function and

$$P_1^* = \frac{2}{9}\frac{\hbar\kappa_h^2\omega_1^4}{\kappa_{h1}\omega_h^3} \quad (19)$$

is the power at which the Lorentzian peak in $|S_{31}|^2$ splits into two. The transmission coefficient $|S_{42}|^2$ and the power P_2^* are given by the same expressions with the interchanged indexes 1 and 2. Analyzing the expression Supplementary Eq. (18), one can show that in the limit $\kappa_h \ll K$ the maxima of the two peaks appearing at $P_1^{\rm in} > P_1^*$ occur at frequencies

$$\omega_\pm = \omega_r \pm \frac{\sqrt{2}\kappa_h}{3}\sqrt{\frac{P_1^{\rm in}}{P_1^*}-1}. \quad (20)$$

Inverting this formula, we find that for a given probe frequency ω the peak in the transmission coefficient occurs at the power

$$P_{\rm peak,1} = P_1^*\left(1+\frac{9(\omega-\omega_r)^2}{2\kappa_h^2}\right). \quad (21)$$

If the signals are applied to both sides of the device, in Supplementary Eq. (18) one should replace the combination $P_1^{\rm in}/P_1^*$ by the sum $P_1^{\rm in}/P_1^* + P_2^{\rm in}/P_2^*$. In Fig. 4 of the main text, we show the powers $P_{\rm peak,1}$ and $P_{\rm peak,2}$ by the white dashed lines. We have used the damping rate $\kappa_h = 1.1$ MHz, which has been obtained by fitting the transmission coefficients (Supplementary Eq. (14)) at low powers and at the flux value $\Phi = 0.5\Phi_0$, at which the data shown in Fig. 4 of the main text have been gathered. The threshold powers $P_1^* = -112$ dBm and $P_2^* = -117$ dBm have been treated as fitting parameters. From the fits we also estimate the anharmonicity as $K/(2\pi) \approx -11.5$ MHz.

Finally, we note that at sufficiently high power the assumption about weak non-linearity of the qubit becomes insufficient, and one should consider full sinusoidal current-phase relation for the three Josephson junctions of the qubit. Here we do not consider this regime. It is well known, however, that in this limit the resonator becomes decoupled from the qubit, and the transmission lines both in $|S_{31}|^2$ and in $|S_{42}|^2$ shift to the bare frequency of the hybrid mode f_h, see Fig. 4 of the main text.

SUPPLEMENTARY NOTE 3

Two tone-spectroscopy:

To determine the qubit transition frequencies we have performed the two-tone spectroscopy as follows. For every value of magnetic flux we choose the probe frequency of a continuous weak microwave signal 'probe tone' (tone one) using a vector network analyzer (VNA). Once the flux specific probe frequency is chosen, using a separate microwave signal generator a 'pump tone' (tone two) is applied to excite the qubit energy levels. The combined results of the one tone and the two tone spectroscopies are presented in Supplementary Fig. 2.

To fit the obtained spectra numerically and to find the coupling constants between the qubit and the resonators, we adapted the following procedure. First, we diagonalize the Hamiltonian of the flux qubit using two dimensional plane waves as described in article [1]. In this way, we find the dependence of the transition frequency $f_{01}(\Phi)$ between the lowest and the first excited states of the qubit decoupled from the resonators on the magnetic flux Φ. Fitting the obtained $f_{01}(\Phi)$ dependence to the results of spectroscopy away from the anti-crossing points, we estimate the parameters of the flux qubit. Namely, we find the asymmetry parameter $\alpha = 0.632$ and the Josephson energies of the two bigger junctions of the SQUID loop, $E_J/(2\pi\hbar) = I_C/4\pi e = 37.5$ GHz. Next, we use the equation describing two coupled oscillators,

$$f_r = \sqrt{\frac{f_i^2+f_{01}^2 \pm \sqrt{(f_i^2-f_{01}^2)^2+16g_i^2 f_i f_{01}}}{2}}, \quad (22)$$

to fit every avoided crossing between the hybrid modes of the resonator (indicated by the index i) and the flux dependent qubit frequency in Supplementary Fig. 2. In the range of frequencies shown there we observe two hybrid resonator modes, low frequency mode (index l) and high frequency mode (index h). The frequencies of these modes are $f_l = 6.027$ GHz and $f_h = 6.762$ GHz, and the corresponding couplings are $g_l/(2\pi) \approx 1$ MHz and $g_h/(2\pi) \approx 175$ MHz. Based on these numbers, from Supplementary Eq. (12) and with the system parameters given in the previous section we can estimate the couplings g_1 and g_2, which appear in the initial Hamiltonian (Supplementary Eq.(2)): $g_1/(2\pi) \approx -89$ MHz and $g_2/(2\pi) \approx 155$ MHz.

SUPPLEMENTARY NOTE 4

Rectification (R) plot with background noise. In Supplementary Fig. 3, for the lowest reported power (-134 dBm), we show the rectification ratio R plot without filtering the background noise ($|S_{42}|^2 + |S_{31}|^2 > 0$).

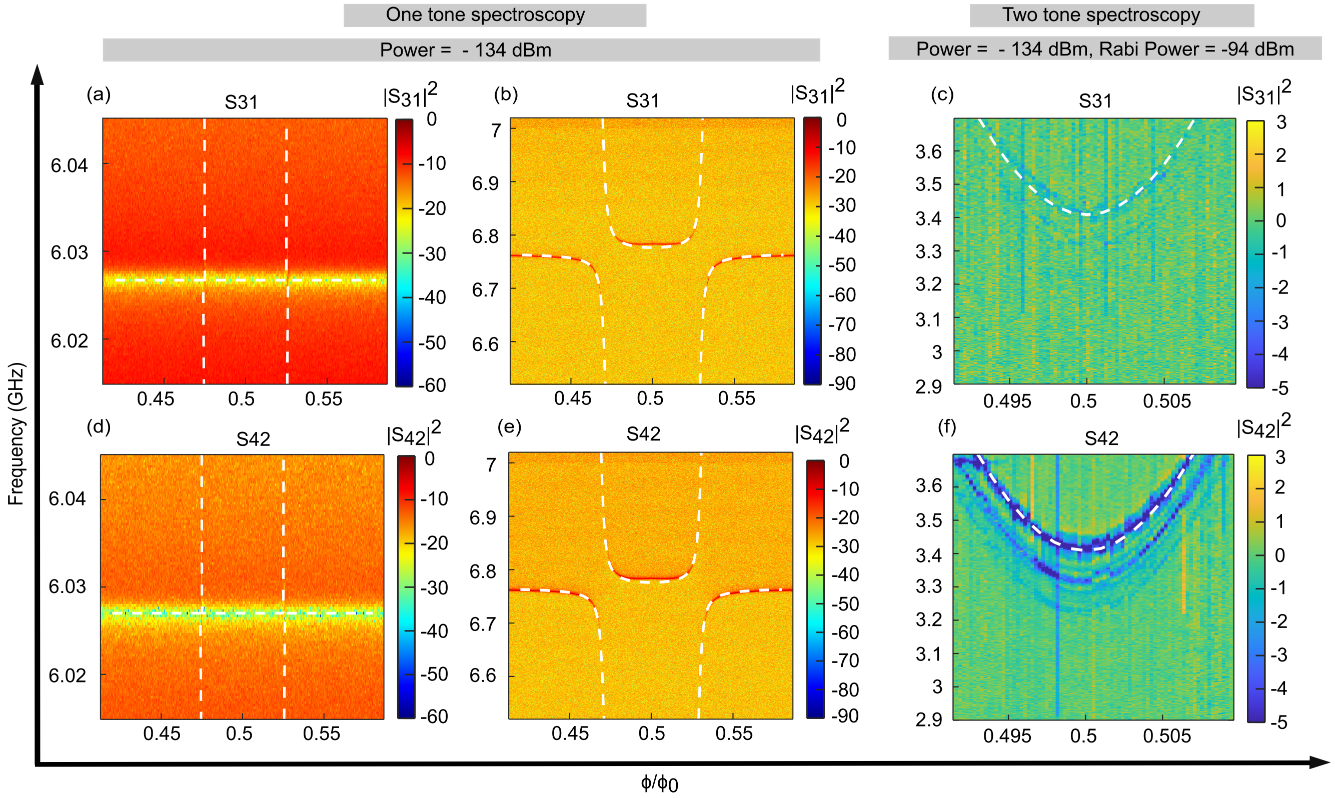

Supplementary Figure 2. One tone and two tone-spectroscopy for -134 dBm input probing power. Panels (a) and (b) show the one tone-spectroscopy data, and panel (c) two tone-spectroscopy data, with probe signal coming through the port 1. Analogously, panels (d) and (e) show one tone-spectroscopy and (f) two tone-spectroscopy, with the probe signal arriving through the port 2. In panels (c) and (f) the pump tone power (Rabi power) is -94 dBm. The frequency step size used in the measurements is 300 KHz. At low input power, the amplitude difference between the maximum resonance point of the transmission to its consecutive frequency point (frequency step size) is much larger than the background noise amplitude. Therefore, the error in determining the frequency will be within ± 300 KHz.

---

[1] Upadhyay, R., Thomas, G., Chang, Y-C., Golubev, D.S., Guthrie, A., Gubaydullin, A., Peltonen, J. T. & Pekola, J.P. Robust Strong-Coupling Architecture in Circuit Quantum Electrodynamics. Phys. Rev. Applied **16**, 044045 (2021).

[2] Abuwasib, M., Krantz, P.& Delsing, P. Fabrication of large dimension aluminum air-bridges for superconducting quantum circuits. Journal of Vacuum Science & Technology B **31**, 031601 (2013).

[3] Janzen, N., Kononenko, M.,Ren, s. & Lupascu, A. Aluminum air bridges for superconducting quantum devices realized using a single-step electron-beam lithography process. Phys. Lett. **121**, 094001 (2022).

[4] Collett, M. J. & C. W. Gardiner. Squeezing of intracavity and traveling-wave light fields produced in parametric amplification. Phys. Rev. A **30**, 1386 (1984).

[5] Segal, D. & Nitzan, A. Spin-Boson Thermal Rectifier. Phys. Rev. Lett. 94, 034301 (2005).

[6] Yamamoto, T., Inomata, K., Koshino, K., Billangeon, P.-M., Nakamura, Y. & Tsai, J. S. Superconducting flux qubit capacitively coupled to an LC resonator. New J. Phys. **16**, 015017 (2014).

[7] Yamaji, T., Kagami, S., Yamaguchi, A., Satoh, T., Koshino, K., Goto, H., Lin, Z. R., Nakamura, Y., & Yamamoto, T. Spectroscopic observation of the crossover from a classical Duffing oscillator to a Kerr parametric oscillator. Phys. Rev. A **105**, 023519 (2022).

[8] Drummond, P. D., and Walls, D. F. Quantum theory of optical bistability. I: Nonlinear polarisability model. J. Phys. A: Math. Gen. **13**, 725 (1980).

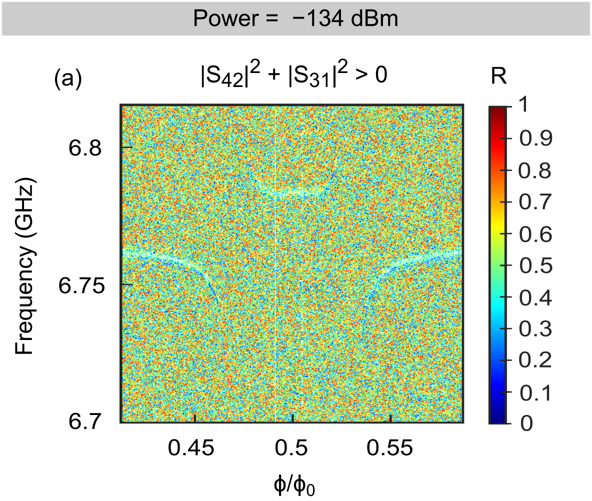

Supplementary Figure 3. The transmission rectification ratio R computed from Eq.1 of the main text, with the noisy background. Estimated error bars are ±35%. More details on error bar estimation are mentioned in Section II of the main text.
